# Triterpenoids in Echinoderms: Fundamental Differences in Diversity and Biosynthetic Pathways

**DOI:** 10.3390/md17060352

**Published:** 2019-06-13

**Authors:** Emily J. S. Claereboudt, Guillaume Caulier, Corentin Decroo, Emmanuel Colson, Pascal Gerbaux, Michel R. Claereboudt, Hubert Schaller, Patrick Flammang, Magali Deleu, Igor Eeckhaut

**Affiliations:** 1 Biology of Marine Organisms and Biomimetics Unit, Research Institute for Biosciences, University of Mons-UMONS, 7000 Mons, Belgium; guillaume.caulier@umons.ac.be (G.C.); patrick.flammang@umons.ac.be (P.F.); igor.eeckhaut@umons.ac.be (I.E.); 2 Laboratory of molecular biophysics of interfaces, Gembloux Agro-Bio Tech, University of Liege, 5030 Gembloux, Belgium; magali.deleu@uliege.be; 3 Organic Synthesis and Mass Spectrometry Laboratory, Interdisciplinary Center for Mass Spectrometry, Research Institute for Biosciences, University of Mons—UMONS, 7000 Mons, Belgium; corentin.decroo@umons.ac.be (C.D.); emmanuel.colson@umons.ac.be (E.C.); pascal.gerbaux@umons.ac.be (P.G.); 4 Department of Marine Science and Fisheries, College of Agricultural and Marine Sciences, Sultan Qaboos University, 123 Al-Khod, Oman; mclaereboudt@mac.com; 5 Institut de Biologie Moléculaire des Plantes du CNRS, Université de Strasbourg, 67084 Strasbourg Cedex, France; hubert.schaller@ibmp-cnrs.unistra.fr

**Keywords:** triterpenoids, saponins, sterols, echinoderms

## Abstract

Echinoderms form a remarkable phylum of marine invertebrates that present specific chemical signatures unique in the animal kingdom. It is particularly the case for essential triterpenoids that evolved separately in each of the five echinoderm classes. Indeed, while most animals have Δ^5^-sterols, sea cucumbers (Holothuroidea) and sea stars (Asteroidea) also possess Δ^7^ and Δ^9(11)^-sterols, a characteristic not shared with brittle stars (Ophiuroidea), sea urchins (Echinoidea), and crinoids (Crinoidea). These particular Δ^7^ and Δ^9(11)^ sterols emerged as a self-protection against membranolytic saponins that only sea cucumbers and sea stars produce as a defense mechanism. The diversity of saponins is large; several hundred molecules have been described in the two classes of these saponins (i.e., triterpenoid or steroid saponins). This review aims to highlight the diversity of triterpenoids in echinoderms by focusing on sterols and triterpenoid glycosides, but more importantly to provide an updated view of the biosynthesis of these molecules in echinoderms.

## 1. Introduction

Echinoderms form a phylum of mostly benthic marine invertebrates, found in a continuous distribution from the intertidal zone to the deepest depths of the ocean. They are a sister group to chordates and are basal deuterostomes [1]. Approximately 7000 extant echinoderm species have been described, falling into five classes: Echinoidea (regular and irregular sea urchins), Holothuroidea (sea cucumbers), Asteroidea (sea stars or starfish), Crinoidea (sea lilies and feather stars), and Ophiuroidea (brittle stars and basket stars), as well as a number of extinct classes known only from the fossil record [2]. Echinoderms feed in a variety of ways; some are suspension feeders (Crinoidea, Ophiuroidea, and dendrochirote Holothuroidea), some are scavengers or even predators (Asteroidea), and the majority of aspidochirote holothuroids are deposit feeders that actively contribute to the bioturbation of sedimentary organic matter and play an important role in the detritus food web by recycling the organic matter and oxygenating the sediment [3,4,5].

A growing number of marine organisms are being chemically investigated in the search for new biomolecules with pharmacological potential [6,7]. This is particularly the case of echinoderms in which each class seems to produce specific metabolites, uncommon in the animal kingdom [8]. Quinonic pigments are specific to Echinoidea (i.e., naphtoquinones; [9,10,11]) and Crinoidea (i.e., anthraquinones; [12]) but are not present in Holothuroidea and Asteroidea, which both produce their specific types of triterpenoid glycosides (i.e., saponins) [13,14]. Ophiuroidea may present a few quinonic pigments but they are the only echinoderm class producing 3α-hydroxysterols [8,15]. Most of these molecules were shown to have a chemical defense role [9,16,17], particularly for sea cucumbers and sea stars that have also developed biosynthetic adaptations to resist their own saponins [18,19].

The triterpenoid composition of invertebrates results from the balance between several contributory sources of sterols. To assess these different sources, several factors need considering: (a) the spectrum of sterols encountered in the diet of the animal and the selectivity which the animal displays for the absorption, or excretion, of any particular compound in the mixture; (b) the assimilation by the host animal of sterols produced by symbiotic microorganisms (algae or other associated organisms, such as bacteria or fungi in the digestive tract); (c) the capacity of an invertebrate to modify absorbed dietary sterols; and (d) the contribution, if any, from de novo biosynthesis of sterol from simple precursors, such as acetyl-CoA and mevalonic acid [20].

Although saponin and sterol diversity and triterpenoid biosynthesis have been investigated as separate research fields, the intimate relationship between these three domains has never been considered. The aim of this review is to highlight the fundamental differences in the diversity of triterpenoids in echinoderms in the form of sterols and triterpenoid glycosides, but more importantly to provide an updated and united view of the biosynthesis of these molecules in echinoderms.

## 2. Sterol Occurrence and Diversity in Echinoderms

Sterols are isopentenyl diphosphate-derived molecules vital for eukaryotic life. They are present in all eukaryotes, where they are essential and are involved in both intra- and intercellular signaling and in the organization of cellular and organelle membranes. In membranes, they affect fluidity and permeability [21,22] and are major contributors to the formation of lipid rafts: Regions of cell membranes characterized by a reduced fluidity formed by the close association of sterols with sphingolipids accommodating embedded functional proteins essential to cell function [23,24,25,26].

Cholesterol and ergosterol are the major sterols accumulating in animals and fungi, respectively. The phytosterols (i.e., C24 alkylsterols), campesterol, stigmasterol, and sitosterol are the most abundant sterols in the plant kingdom [27].

After the pioneering efforts of Bergmann and colleagues in 1943 [28], it was recognized that the sterol patterns in marine invertebrates reflect the diversity of mixtures of sterols arising from complex food chains [20]. In the same species, the sterol fractions showed different profiles depending upon the location where the organisms had been collected. Moreover, the ability of further biochemical modification of the dietary sterols by the organisms or their symbionts makes the sterol mixtures even more complex. Many sterols of unprecedented structures have been isolated from marine sources [29]. Bergmann and his colleagues demonstrated that marine invertebrates in many cases contain complex sterol mixtures consisting of C27, C28, and C29 sterols of varying degrees of unsaturation [30,31]. The sterols found in the phylum, Echinodermata, have proven of particular interest not only from the point of view of their unusual structure, composition, and biological origin but also from phylogenetic considerations.

**Figure 1:**
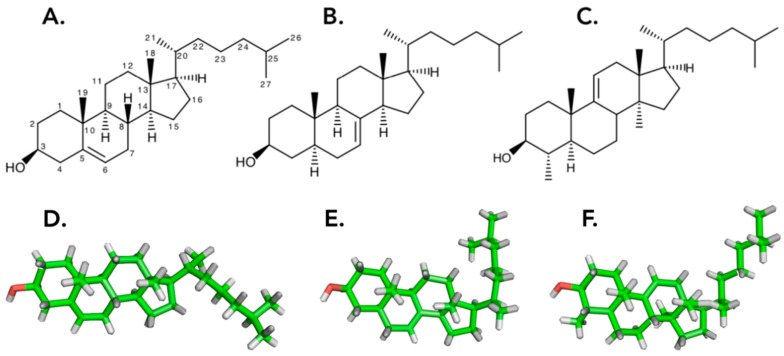
**A**. Structure of cholesterol (with carbon numbers and stereo position of bonds), the primary sterol in most metazoan cell membranes. **B**. 5α-Cholest-7-en-3β-ol, the most abundant free sterol of *Holothuria scabra*. **C**. 4α,14α-dimethyl-5α-cholest-9(11)-en-3β-ol, the second most abundant free sterol in *H. scabra* [36]. **D**,**E**,**F**. 3D structure of the sterols [19].

Considerable research has been conducted on sterols, sulphated sterols, polyhydroxsterols, and steroidal/triterpenoid glycosides (saponins) in Ophiuroidea, Asteroidea, and Holothuroidea [31,32,33], however, very few publications have addressed Crinoidea and Echinoidea [8] (Supplementary data: Table S1). Researchers were quick to realize that a striking feature of the phylum is the dichotomy between Crinoidea and Echinoidea, which contain Δ^5^-sterols (such as cholesterol) (Figure 1A) like most metazoans, while the Asteroidea and Holothuroidea contain complex mixtures of sterols in which molecules with a Δ^7^ double bond are predominant [31,34]. Interestingly, the same dichotomy also appears in the production of saponins, as only the classes of holothuroids and of asteroids synthesize saponins [13,14,35]. Ophiuroidea may be considered as chemically marginal in the phylum of echinoderms as they share some chemical characteristics with both crinoids and echinoids, and some even with asteroids and holothuroids [8]. In addition, ophiuroids have the unique particularity of having a majority of 3α-hydroxysterols (80% 3α, 20% 3β), both free and sulphated [8]. In contrast, Echinoidea sterol composition remains fairly conventional, with over 70% being cholesterol [8]. Concerning Crinoidea, little is known, and future research should focus on increasing the available data for this class of echinoderms [8].

There is a greater structural variety in sterols observed in Asteroidea than in Ophiuroidea, be it in relation to the hydroxy or sulphate groups, or the different insaturation possibilities in the tetracyclic system (Δ0, Δ5, Δ7, Δ9(11)) and the lateral chains [8]. The principal characteristic of Asteroidea sterols is however the predominance of Δ7 sterols in relation to Δ5 sterols.

The free sterol composition of holothuroid tissue [36] also exhibits a large diversity of new and rare sterols (between 70 and 80 structures) [37]. The relative abundances of Δ^7^:Δ^9(11)^:Δ^0^:Δ^5^ compounds were found to be approximately 4:2:1.5:1 [37]. The most abundant Δ^7^ sterol being 5α-Cholest-7-en-3β-ol (Figure 1B), with approximately 15% of the free sterol fraction of the body wall extract of *H. scabra*, followed by the Δ^9(11)^-sterol 4α,14α- dimethyl-5α-cholest-9(11)-en-3β-ol with 13.4% (Figure 1C) [36]. 

Popov et al. (1983) [18] hypothesized that the evolutionary replacement of Δ^5^-sterols with 5α-cholest-7-en-3β-ol and 4α,14α-dimethyl-5α-cholest-9(11)-en-3β-ol, or other unusual sterols in sea cucumbers, mitigates the lytic action of the saponins (triterpenoid glycosides) of the similar structure they produce [18] as part of their defense mechanism. It may also be suggested that a high percentage of toxic saponins in *Holothuria spp.* influences steroidogenesis and stimulates the de novo biosynthesis of unusual sterols [38].

Investigations of the biophysical properties of interactions between sterols and triterpenoid glycosides indeed support this theory and strongly suggest that the replacement of cholesterol by biosynthetic precursors, such as Δ^7^ sterol in the cell membranes of sea cucumbers, allows these organisms to tolerate the presence of their own cytotoxic saponins [19]. This tolerance is notably due to the 3D “L” shaped conformation of these sterols, in comparison to the fairly linear cholesterol. The difference in the 3D conformation of these holothuroid sterols results in different interactions with membrane lipids and a contrasting behavior with holothuroid and non-holothuroid cell membranes in the presence of saponins [19].

Although Asteroidea and Holothuroidea sterol mixtures appear unique in the animal kingdom, and likely in relation with the synthesis of their defensive saponins, much remains to be investigated in terms of the evolutionary appearance of Δ^7^ sterols in these two classes of echinoderms.

## 3. Saponin Occurrence and Diversity in Echinoderms

Saponins form an important class of natural products first discovered in higher plants [39]. In the marine environment, saponins are secondary metabolites mainly produced by echinoderms [40], although saponins have also been isolated from other marine invertebrates, such as octocorals or sponges [41,42,43]. Numerous studies have been conducted on these compounds that are characterized by a large chemical diversity and a wide variety of pharmacological activities [7,39,44,45,46,47,48].

In Echinoderms, saponins have been found exclusively in the classes Holothuroidea (sea cucumbers), in the form of triterpenoid glycosides [44,45,49], and in Asteroidea (starfishes), in the form of steroidal glycosides [46,47,50,51]. Triterpenoid and steroid saponins are derived from the linear 30 carbon precursor, 2,3-oxidosqualene [52]. Both the triterpenoid and steroidal aglycone backbones are isoprenoids that are synthesized from isopentenyl diphosphate (IPP) and dimethylallyl diphosphate (DMAPP) units generated by the mevalonate (MVA) pathway [52] (see next section). 

**Figure 2:**
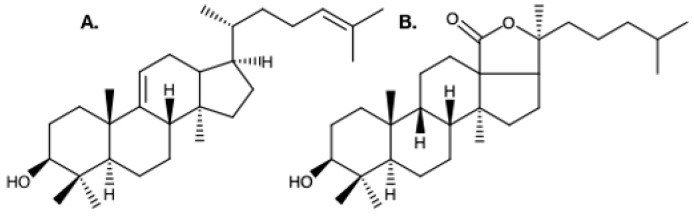
**A**. Structure of the hypothesized precursor of holothuroid triterpenoids; parkeol. **B**. The most abundant aglygone moiety in holothuroid triterpene glycosides; holostanol [53,60].

In sponges and starfishes, the 2,3-oxidosqualene at the origin of the saponin biosynthesis is cyclized into lanosterol which gives way to steroidal saponins. However, in Holothuroidea, the 2,3-oxidosqualene is thought to be cyclized into parkeol, which is then rearranged to form the most abundant holothuroid aglycone, the holostanol [13] (Figure 2). In sea cucumbers, there is only one oligosaccharide attached to the aglycone [53], whereas plant saponins may contain one, two, or three saccharide chains, with a few having an acyl group bound to the sugar moiety [54]. The oligosaccharide moiety of holothuroid saponins, which can be sulfated or non-sulfated, can contain up to six sugar units consisting mainly of glucose, 3-O-methylglucose, quinovose, and xylose [55,56]. Saponin profiles differ as a function of the species [13], of the body component [49,57,58], the sex [56], and the maturity of the individual [59], both qualitatively (different saponin mixtures) and quantitatively (different relative concentrations).

**Figure 3:**
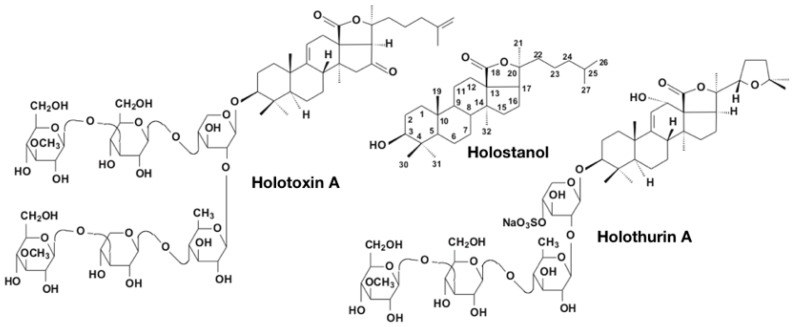
Structures of Holostanol characterized by an 18(20)-lactone fragment. Most sea cucumber triterpene glycosides possess such a type of holostane aglycones. Holotoxin A1 is an example of non-sulfated holostane glycoside. Holothurin A is an example of sulfated holostane glycoside [60].

Triterpene glycosides also have some taxonomic specificity for different species and genera of sea cucumbers and even for taxa at the supra-genus level [61,62]. These holothurian glycosides have quite complicated structures and can be distinguished by several independent characteristics: The type and number of monosaccharide units in the carbohydrate chain, the number and positions of sulfate groups attached to monosaccharide units (Figure 3), the position of double bonds in the cyclic system of the aglycone, the number and position of double bonds in the side chain of the aglycone, and the number and different position of hydroxy-, epoxy-, acetyl-, and oxo- groups in the aglycone, etc. [53]. So far, over 700 saponins have been described in Holothuroidea [54]. The majority of the known sea cucumber glycosides possess a 18(20)-lactone ring in the aglycones and form the so-called holostane aglycones. The aglycones preferably have a 7(8)- or 9(11)-double bond and a chain of monosaccharide units usually including d-xylose, d-glucose, d-quinovose, d-3-O-methyl-xylose, and 3-O-methyl-d-glycose [63] (Figure 3). 

Lanostane derivatives without the lactone ring or containing a 18(16)-lactone fragment instead of the 18(20)-lactone belong to the so-called non-holostane glycosides. The aglycones of most sea cucumber saponins belong to the holostane series and only about 40 or so of the described glycosides are non-holostane derivatives (i.e., they do not have an 18(20)-lactone fragment in their aglycone moiety) [53,60,64].

The discovery of non-holostane glycosides have allowed a better understanding of the origin and biosynthetic pathways leading to holostane derivatives. Recently, Kalinin et al. [60] suggested that these non-holostane glycosides were probable evolutionary ancestors and, in many cases, the biosynthetic precursors of holostane glycosides. An evolutionary transition from non-holostane to holostane derivatives may be explained by stronger membranolytic activities of holostane glycosides that presumably allow sea cucumbers to use these natural products as a more effective chemical defense against predators [60].

**Figure 4:**
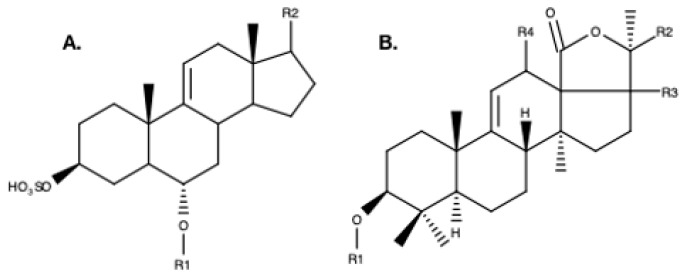
Example structures of **A.** Steroid aglycone saponin backbone from the sea star, *Asterias rubens* [14] **B.** Triterpene aglycone backbone from the sea cucumber, *Holothuria scabra* [13]. R1 represents the position of the glycoside moiety of both saponin types. The position of R2, R3, and R4 can vary and are illustrated here as examples.

Unlike holothuroid saponins, which are triterpenoid glycosides [49], asteroid saponins are steroid glycosides [65,66,67] (Figure 4). Since the 1960s, an investigation of nearly 100 starfish species collected in all climatic areas has led to the identification of some 400 steroid glycosides [68,69]. Three categories of saponins have been identified in sea stars, i.e., polyhydroxysteroid glycosides, asterosaponins, and macrocyclic saponins [66,70]. The asterosaponins, occurring in almost all starfish species, possess well defined structural characteristics. They invariably contain a Δ^9(11)^-3β, 6α-dihydroxysteroidal nucleus with a sulfate residue at C3 and often an oxo substituent at C23 on the aglycone side-chain [35]. The carbohydrate moiety is bound at the carbon atom C6 on the aglycone and includes five to six sugar residues. The most common monosaccharides are β-d- fucopyranose, β-d-quinovopyranose, β-d-xylopyranose, β-d-galactopyranose, and β-d-glucopyranose; 6-deoxy-xylo-hex-4-ulose (DXHU) and α-L-arabinopyranose are less frequently present [50]. In *Asterias rubens*, only asterosaponins have been observed [14,50]. Asterosaponins are pentaglycoside or hexaglycoside sulfated steroids that have high molecular weights (±1200 Da) (Figure 4).

Considering both asteroids and holothuroids and based on observations of organ-specific saponin contents [14,50] and behavioral experiments [71], it has been suggested that saponins could contribute to digestion [70,72], reproduction (e.g., spawn synchronisation) [73,74], as well as intra or interspecific chemical signaling [17,71,75,76,77]. 

## 4. Triterpenoid Biosynthesis

The large diversity of sterols present in the five classes of echinoderms, in addition to the high diversity of saponin aglycone structures in Asteroidea and Holothuroidea, raise interesting questions regarding the source of these triterpenoids. Although there are reports of biosynthesis [78] of triterpenoids in these two classes of echinoderms, what proportion is synthesized de novo and what proportion is directly or indirectly extracted from the diet and then modified by the animal are unknown. Moreover, whether the sources of these triterpenoids are similar across echinoderm classes is yet to be determined. 

Sterols and triterpenes share a common biosynthetic precursor: 2,3-oxidosqualene. This molecule is one of the products of the mevalonate (MVA) pathway that takes place in the cytosol of cells [26,79,80,81,82].

**Figure 5:**
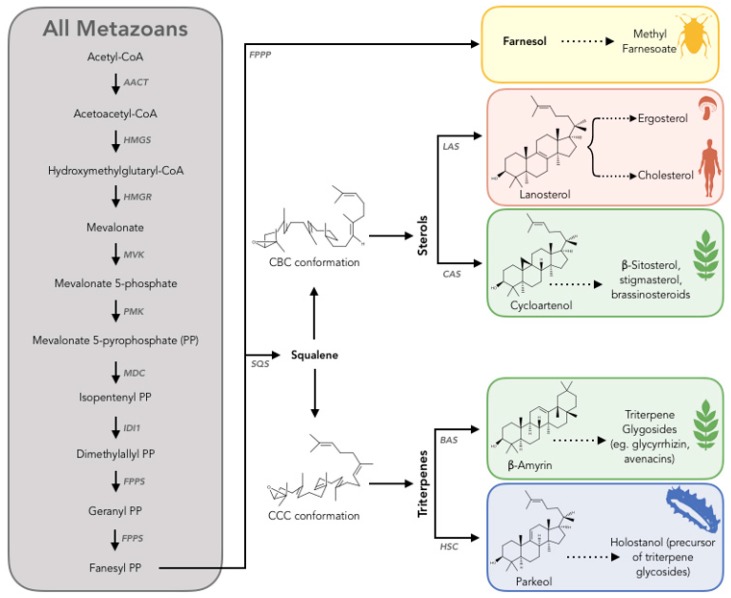
Scheme to summarize the current understanding of triterpenoid biosynthesis in metazoans. The metazoan mevalonate (MVA) pathway according to the published literature is common to all metazoans (gray box) [84]. The subsequent biosynthetic routes to sterols and triterpenes are group dependent. Enzyme abbreviations: AACT, acetoacetyl- CoA thiolase; HMGS, hydroxymethylglutaryl-CoA synthase; HMGR, hydroxymethylglutaryl-CoA reductase; MVK, mevalonate kinase; PMK, phosphomevalonate kinase; MDC, mevalonate-5-decarboxylase; IDI1, Isopentenyl diphosphate isomerase; FPPS, fanesyl diphosphate synthase; SQS, squalene synthase; FPPP, fanesyl diphosphate phosphatase; LAS, lanosterol synthase; CAS, cycloartenol synthase; BAS, β-amyrin synthase; HSC, holothuroid squalene cyclase. Other abbreviations: CBC, chair-boat-chair; CCC, chair-chair-chair [83].

Mevalonate synthesis from acetyl-CoA is conserved in all metazoans (Figure 5, gray box). The final steps of the pathway display group-specific variations: Cholesterol synthesis in vertebrates (Figure 5, orange box) or methyl farnesoate synthesis in arthropods (Figure 5, yellow box) [83]. Once synthesized, 2,3-oxidosqualene can be cyclized by a series of oxidosqualene cyclases (OSCs) to produce sterols: Lanosterol synthase (LAS) in fungi and most metazoan, and cycloartenol synthase (CAS) or triterpenes, e.g., β-amyrin synthase (BAS) in plants (Figure 5, orange, green, and blue boxes).

Studies on echinoderm triterpenoids and in particular their biosynthesis came in two major waves of publications one from the mid 1960s to 1980s that primarily used in vivo radio-labeled feeding or injection experiments (e.g., [20,73,85,86]), and a second one is currently taking place using next generation sequencing (e.g., [51,87,88,89]).

The use of ^14^C-labeled acetate, mevalonate, or cholesterol during feeding experiments confirmed the existence of the MVA pathway in echinoderms [85,86]. Injections of labeled (2-^14^C) mevalonic acid and (4-^14^C) cholesterol into the starfish, *Marthasterias glacialis*, revealed the synthesis of steroidal saponins both de novo and from dietary sterols, although the authors admitted that levels of incorporation were low, particularly for cholesterol [73].

De novo biosynthesis of triterpene glycosides in sea cucumbers was also observed in radiolabeling experiments [90,91]. The radiolabeled acetate and mevalonate were used as precursors for 2,3-oxidosqualene de novo synthesis, which was then usually transformed into lanosterol and parkeol (lanosta-9(11), 24-dien-3b-ol) after cyclization [86,92,93] and also concluded that “all echinoderm classes can form at least some sterols from acetate and mevalonate via the MVA pathway”. However, the authors of [85], through an analysis of the sterol composition and metabolism, suggested that most sterols occurring in echinoderms were formed by transforming exogenous (i.e., dietary) Δ^5^ sterols. These authors also postulated that there were divergent biosynthetic pathways at the 4,4-dimethyl sterol level, leading either to the Δ^7^-sterols or to steroidal saponins [85]. However, this remains only a hypothesis.

Although both [85] and [73] established that the sea star *Asterias rubens* was capable of at least limited de novo synthesis of cholesterol from mevalonic acid [85], later reports contradicted these initial findings and stated that “it is generally accepted that sea cucumbers and sea stars cannot perform the final steps in the biosynthesis of cholesterol, in other words that they are unable to introduce the double bond at C5 and to saturate the double bond at C7” [94].

**Figure 6:**
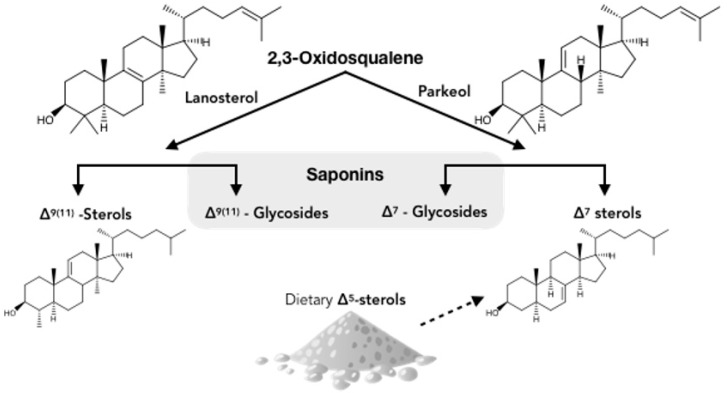
Hypothetical scheme of biosynthesis (solid arrows) and metabolism (dashed arrow) of steroids and triterpenoids in sea cucumbers. Adapted from Stonik et al. (1999) [96].

According to the hypothetical scheme proposed by Makarieva et al. [95] (Figure 6), there are several possible routes of steroid metabolism in holothurians. The first involves the de novo biosynthesis of 14α-methylcholest-9(ll)-en-3β-ol. Dietary sterols (especially C27-Δ^5^-sterols) are sulfated or transformed into stanols and Δ^7^-sterols while other free sterols are converted into sterol xylosides [95,96]. This results in complex cellular membranes in holothurians which may render cells resistant to the action of triterpene saponins as observed [18,19]. The authors go on to speculate that in sea cucumbers, the biosynthesis of saponins is an ancient anabolic pathway and that the formation of Δ^9(11)-^sterols from parkeol may have appeared later as a response to the endo-toxicity of the saponins. However, again this is only a hypothesis that remains to be investigated.

Overall, it would seem that there are conflicting conclusions in the older literature in terms of the origin of precursors and the possibility of de novo synthesis of triterpenoids in echinoderms. Goad et al. [85] attribute these inconsistencies to incomplete experimental design around the use of isotopes. Indeed, the technique requires that either all the components of the complex sterol mixture are labeled or alternatively if only one sterol of the mixture is labeled this must co-crystalize with the other components of the mixture but not be lost by fractional crystallization. If one of these requirements is not met, it is possible that a continuous drop in specific radioactivity will result during several re-crystallizations and this could be interpreted as a lack of incorporation of radioactivity into the sterols [85]. In addition, the possible participation of the microbiome of these marine invertebrates in some of the triterpenoid metabolic pathways has not been considered.

In more recent years, the use of isotope-labeled precursors has lost popularity, and genomic and transcriptomic analyses have proven to be a popular and powerful tool for the investigation of biosynthetic pathways [51,87,88,89]. Recent work has mainly focused on holothuroids, rather than other echinoderm classes, so gaps in the knowledge regarding the MVA pathway across the echinoderm phylum remain. Overall, the current knowledge about saponin biosynthetic pathway(s) has come mostly from plant studies [78], and how sea cucumbers gained the ability to synthesize saponins, evolutionary speaking, remains a source of intrigue.

**Figure 7:**
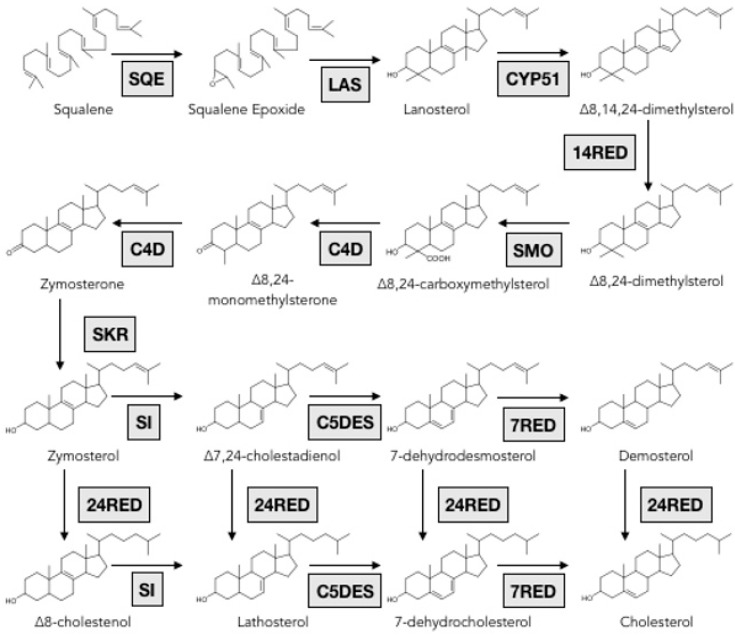
Scheme of the post squalene pathway for cholesterol biosynthesis. SQE: Squalene epoxydase; LAS: Lanosterol synthase; CYP51: Lanosterol-14α-demethylase; 14RED: Sterol-14-reductase; SMO: Sterol-4α-methyl-oxidase; C4D: C4 decarboxylase; SKR: sterone ketoreductase; SI: Sterol-8-isomerase; C5DES: Sterol-C5-desaturase; 24RED: Sterol-24-reductase; 7RED: Sterol-7-reductase. Reproduced with permission from Marijanovic et al., Molecular Endocrinology, published by Oxford University Press (2003), enzymes names were simplified.

Genes involved in the formation of triterpene backbones in echinoderms are rarely reported, however, biosynthetic pathways of triterpenes were unraveled in other organisms and biosynthetic enzymes are functionally well characterized [88,97,98]. In the animal kingdom, once the mevalonate pathway synthesizes squalene, the linear hydrocarbon chain is cyclized into lanosterol by an oxidosqualene cyclase (OSC) followed by various enzymes that tailor the four-ring molecule to eventually form cholesterol (Figure 7).

Cyclization is a critical step in triterpenoid biosynthesis. In some sea cucumbers, in contrast with most animals, 2,3-oxidosqualene is cyclized into parkeol instead of the isomeric lanosterol [92,93]. Parkeol could be transformed into glycosides both in in vivo and in vitro experiments with the Cuvierian tubules of the sea cucumbers, *Holothuria floridana* and *Actinopyga agassizi* [78]. ^3^H-labeled lanosterol, on the other hand, was incorporated into aglycone moieties of saponins in *Stichopus californicus* [99], which was also confirmed in *Eupentacta fraudatrix* [85]. However, no radiolabeled glycosides were detected in *Bohadschia argus* and *Holothuria mexicana* when ^3^H-labeled lanosterol or parkeol were supplied [92,93]. 

Nevertheless, these puzzling results showed that different groups of saponins with either Δ^9(11)^ or Δ^7(8)^-unsaturations in their aglycones can be formed via cyclization of oxidosqualene into either lanosterol or parkeol (or even into lanosta-7,24-dien-3β-ol) [100]. Whether lanosterol and parkeol are eventually incorporated into saponin remains somewhat controversial [100]. Yet, these two compounds are nevertheless important intermediates for the synthesis of aglycones containing 18(20)-lactone [88].

In an analysis of the transcriptomic data of the holothuroid, *Stichopus horrens*, all the genes involved in the MVA pathway were found [88]. However, the authors pointed out that the results for the post-squalene pathway become quite complicated and are sometimes contradictory to those obtained from earlier radiolabeling experiments. The high expression of one oxydosqualene cyclase, OSC1, in intestinal tissue, which also showed a significantly high content of saponin, suggested a possible link between this OSC1 and saponin production [88].

**Figure 8:**
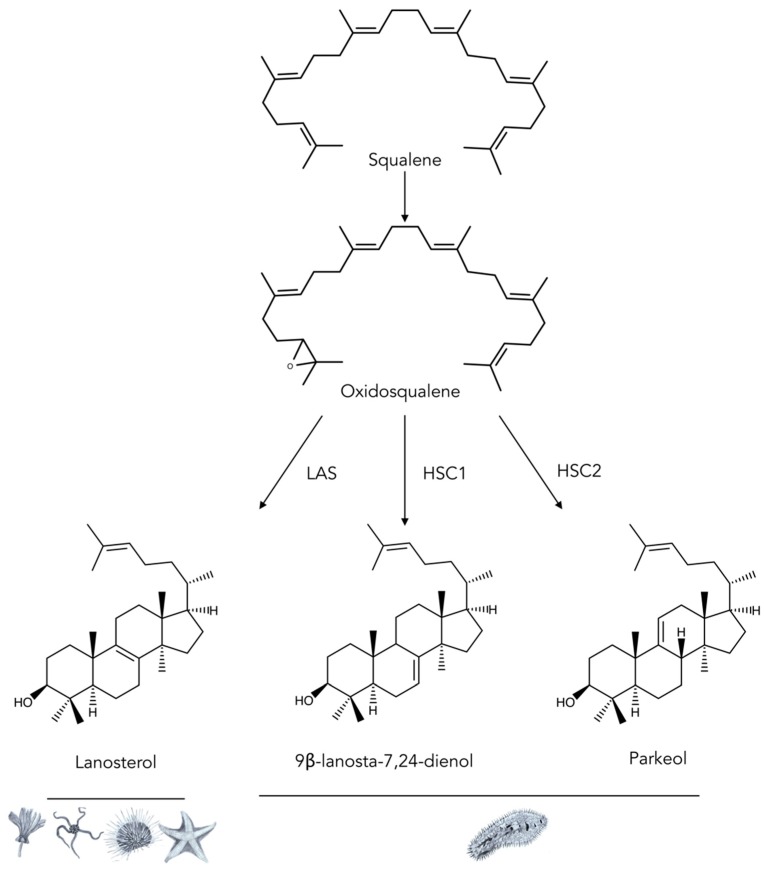
Summary of the diversity of oxido-squalene cyclases (OSCs) in echinoderms. LAS: Lanosterol squalene cyclase is the principal cyclase of the animal kingdom. HSC1 and HSC2: Holothuroid squalene cyclases 1 and 2 are recently discovered cyclase isoforms identified in *S.* *horrens* [88] and *A. japonicus* [89]. In order to unify nomenclature across the literature, holothuroid OSCs were labeled HSC (instead of LAS [104] or OSC [105]). Pictograms underneath the structure illustrate the five classes of echinoderms: Crinoidea, Ophiuroidea, Echinoidea, Asteroidea, and Holothuroidea.

The two OSCs identified in *S. horrens* present the classical DCTAE peptide motif previously identified in lanosterol synthase. However, sequence variability between these two sea cucumber OSCs implies that they may have different catalytic function [88]. In fact, a novel parkeol synthase identified in rice, *Oryza sativa,* contained a similar DCTATE motif [101]. The triterpene glycosides identified in *S. horrens* possess a Δ^7(8)^-double bond in their aglycone moieties [102]. It is thus possible that 2,3-oxidosqualene could be catalyzed into lanosta-7(8),25(26)-dien-3β-ol by OSC1 and OSC2 (Figure 8), which could then be directly used in the biosynthesis of the aglycones. The authors conclude that further studies are needed to characterize the functionality of these two genes [88]. In 2018, Li et al. [89] also found two predicted OSC genes (named in their study *LAS1* and *LAS2*) in the genome of *Apostichopus japonicus*. Evolutionary analysis suggested that these LAS genes showed high evolutionary rates in sea cucumbers in comparison to other animal groups and contained many plant-like motifs that were not present either in sea urchins or in starfish. A functional analysis of yeast expressing the *LAS1* and *LAS2* from *A. japonicus* revealed that the cyclization of 2,3-oxidosqualene enhanced the production of parkeol (LAS1) and 9β-lanosta-7, 24-dienol (*LAS2*) instead of the expected lanosterol [89]. Parkeol has previously been suggested to be the triterpene precursor of saponins in sea cucumbers [78]. Whether 9β-lanosta-7,24 dienol is also a saponin precursor remains to be determined.

Li et al. (2018) investigated the integrity of the post squalene pathway for the route of cholesterol synthesis in animals in the genome of the sea cucumber, *A. japonicus*. They found that two genes, *Cyp51* (lanosterol-14α-demethylase) and *Dhcr7* (7-Dehydrocholesterol reductase), were absent in the sea cucumber genome. This is in contrast to the observation of the full gene sets in the sea urchin, *Strongylocentrotus purpuratus*, and the starfish, *Acanthaster planci*, suggesting that the sea cucumber, *A. japonicus*, may have lost the ability to synthesize cholesterol de novo [89], which is consistent with previous observations of extremely low cholesterol levels in sea cucumbers [36,103]. In addition, the absence of Cyp51 (i.e., lanosterol-14α-demethylase) in the sea cucumber genome also supports the previous speculation that the blockage of C-14 demethylation leads to the accumulation of 14α-methylated Δ^9(11)^-sterols in cell membranes of sea cucumbers [18,89,96], contributing to resistance to their own toxins [19].

These two studies suggest that the extraordinary ability of sea cucumbers to synthetize saponins, and cell membrane sterols that mitigate the cytotoxicity of saponins, is enabled by a modification of the lanosterol synthase, which possibly occurred through convergent evolution [88,89] This also implies that even for very complex metabolic pathways, modifying just one key gene can lead to the generation of a new adaptive trait in an organism. Interestingly, however, this does not seem to be the case in sea stars [89].

## 5. Conclusions

Echinoderms, ubiquitous in the marine environment, are important from evolutionary, ecological, and socioeconomic perspectives. Together with protochordates, chordates, and hemichordates, they form the deuterostome clade, making them a crucial node in the study of chordate ancestry [106]. Echinoderms are also a rich source of pharmacologically active molecules. Triterpenoids are among the most abundant of these, therefore understanding their diversity and biosynthesis remains a fundamental area of research. As analytical techniques evolve, the detection and quantification of new triterpenoids in these marine invertebrates has been more and more accessible by laboratories around the world, in a wide range of species. In addition, as more genetic data becomes available, investigating metabolomes using in silico techniques (genomics, proteomics, and transcriptomics) has also become popular in recent years. However, knowledge gaps still remain, in particular the homeostasis of the sterol content acquired from both dietary sources and de novo synthesis. In this paper, we provided an updated view on the biosynthesis of triterpenoids in echinoderms. This is particularly in the cases of sea stars and sea cucumbers who have altered their sterol content to include primarily Δ^7^-sterols versus the ubiquitous Δ^5^-sterols in the other echinoderm classes and in the animal kingdom in general. There seems to be an intimate relationship between the synthesis of these Δ^7^-sterols and saponins, both triterpenoidal in sea cucumbers and steroidal in sea stars. Linking mid-20th century research using isotopes with today’s genomic research with modern analytical techniques will surely shine light on the homeostasis of triterpenoids in echinoderms.

## Supplementary Materials

The following are available online at https://www.mdpi.com/1660-3397/17/6/352/s1, Table S1: Compiled list of echinoderm species for which the sterol content has been completely or partially described.
