# Supplementary material for: Triterpenoids in Echinoderms: Fundamental Differences in Diversity and Biosynthetic Pathways
Source: Mar Drugs. 2019 Jun 13;17(6):352. doi: 10.3390/md17060352 (PMC6627624; doi:10.3390/md17060352)
Supplement: Supplementary file 1 [file marinedrugs-17-00352-s001.pdf]

Supplementary data

Table 1. Compiled list of echinoderm species for which the sterol content has been completely or partially described.

Holothuroidea

Species	Reference	Species	Reference
Benthodytes lingua	[105]	*Trochostoma orientale*	[36]
Stichopus tremulus	[105]	*Bathyplotes natans*	[36]
Mesothuria verrilli	[105]	*Abyssocucumis abyssorum*	[106]
Pseudostichopus trachus	[36]	*Oneirophanta mutabilis*	[106]
Holothuria nobilis	[36]	*Peniagone vitrea*	[106]
Holothuria scabra	[36]	*Protankyra brychia*	[106]
Holothuria atra	[34]	*Cucumaria planci*	[107]
Cucumaria elongata	[85]	*Holothuria tubulosa*	[107,108]
Cucumaria hydriani	[85]	*Stichopus japonicus*	[108,109]
Cucumaria lactea	[110]		
Stichopus regalis	[107]		

Ophiuroidea

Species	Reference	Species	Reference
Ophiacantha sp.	[106]	*Ophioplocus januarii*	[111]
Ophiura bathybia	[106]	*Ophionotus victoriae*	[111]
Ophiocomina nigra	[110]	*Ophioderma longicauda*	[112]
Ophiura albida	[85,107]	*Ophiocoma wendtii,*	[111]

Ophiocoma insularia	[34]	*Ophiocoma echinata*	[111]

Echinoidea

Species	Reference	Species	Reference
Echinothrix diadema	[34]	*Echinus acutus*	[113]
Anthocidaris crassispina	[114]	*Echinus esculentus*	[115]
Astriclypeus manni	[114]	*Paracentrotus lividus*	[113,116]
Echinocardium cordatum	[114]	*Psammechinus miliaris*	[113]
Temnopleurus toreumaticus	[114]	*Scaphechinus mirabilis*	[114]

Crinoidea

Species	Reference	Species	Reference
Antedon sp.	[34]	*Comatula sp.*	[117]
Antedon bifida	[110]		

Asteroidea

Species	Reference	Species	Reference
Asterias rubens	[85,94,118]	*Asterias amurensis*	[119-121]
Acanthaster planci	[34]	*Asterina pectinifera*	[119-121]
Ctenodiscus crispatus	[122]	*Astropecten aurantiacus*	[112]
Culcita schmideliana	[123]	*Astropecten irregularis*	[85]
Distolasterias sticantha	[120]	*Astropecten polyacanthus*	[119]
Echinaster sepositus	[112]	*Astropecten scoparius*	[119]
Henricia sanguinolenta	[85]	*Certonardoa semiregularis*	[120,121]

Leiaster leachii	[86,124]	*Coscinasterias acutispina*	[86,119]
Luidia ciliaris	[85]	*Linckia multifora*	[123]

107. Ballantine, J.; Lavis, A.; Morris, R. Marine sterols. XV. Sterols of some oceanic holothurians. *J. Exp. Mar. Biol. Ecol.* **1981**, *53*, 89–103.
108. Nomura, T.; Tsuchiya, G.; Andre, D.; Barbier, M. Sur les fractions insaponifiables des holothuries, *Stichopus japonicus* et *Holothuria tubulosa*. *Bull. Jap. Soc. Sci. Fish* **1969**, *35*, 293–298.
109. Toyama, Y.; Takagi, T. Fatty oils of aquatic invertebrates—X. Fatty oils of *Stichopus japonicus*, *Astriclypeus manni*, *Clypeaster japonicus*, and *Gorgonocephalus caryi*. *Nippon Kagaku Zasshi* **1956**, *77*, 102–105.
110. Rubinstein, I. *A Study of Marine Sterols*; University of Liverpool: Liverpool, UK, 1973.
111. Duque, C.; Rojas, J.; Zea, S.; Roccatagliata, A.J.; Maier, M.S.; Seldes, A.M. Main sterols from the ophiuroids *Ophiocoma echinata*, *Ophiocoma wendtii*, *Ophioplocus januarii* and *Ophionotus victoriae*. *Biochem. Syst. Ecol.* **1997**, *25*, 775–778.
112. Voogt, P.A. Sterols of Some Echinoids. *Arch. Int. Physiol. Biochim.* **1972**, *80*, 883–891, doi:10.3109/13813457209070439.
113. Yasuda, S. Sterol compositions of echinoids (sea urchin, sand dollar and heart urchin). *Compar. Biochem. Physiol. Part B Compar. Biochem.* **1974**, *49*, 361–366, doi:10.1016/0305-0491(74)90171-0.
114. Smith, A.G.; Goad, L.J. Sterol biosynthesis by the sea urchin *Echinus esculentus*. *Biochem. J.* **1974**, *142*, 421–427.
115. Salaque, A.; Barbier, M.; Lederer, E. Sur la biosynthèse des stérols de l'huître (*Ostrea gryphea*) et de l'oursin (*Paracentrotus lividus*). *Compar. Biochem. Physiol.* **1966**, *19*, 45–51.
116. Bolker, H.I. Crinosterol: A Unique Sterol from a Comatulid Crinoid. *Nature* **1967**, *213*, 905–906, doi:10.1038/213905a0.
117. Smith, A.G.; Rubinstein, I.; Goad, L.J. The sterols of the echinoderm *Asterias rubens*. *Biochem. J.* **1973**, *135*, 443–455, doi:10.1042/bj1350443.
118. Matsuno, T.; Nagata, S.; Mizutani, K. Sterols of Starfishes-II. *Nippon Suisan Gakkaishi* **1972**, *38*, 144–147.
119. Kobayashi, M.; Tsuru, R.; Todo, K.; Mitsuhashi, H. Asteroid sterols. *Tetrahedron Lett.* **1972**, *13*, 2935–2938, doi:10.1016/S0040-4039(01)84976-5.
120. Kobayashi, M.; Mitsuhashi, H. Marine sterols—IV: Structure and synthesis of amuresterol, a new marine sterol with unprecedented side chain, from *Asterias amurensis* lütken. *Tetrahedron* **1974**, *30*, 2147–2150.
121. Grossert, J.S.; Mathiaparanam, P.; Hebb, G.D.; Price, P.; Campbell, I.M. The sterols of the echinoderm,*Ctenodiscus crispatus* retzius. *Experientia* **1973**, *29*, 258–259, doi:10.1007/BF01926460.
122. Sheikh, Y.M.; Kaisin, M.; Djerassi, C. Steroids from starfish. *Steroids* **1973**, *22*, 835–850.
123. Teshima, S.-i.; Kanazawa, A. Biosynthesis of sterols in a starfish, *Laiaster leachii*. *Compar. Biochem. Physiol. Part B Compar. Biochem.* **1975**, *52*, 437–441, doi:10.1016/0305-0491(75)90158-3.
